# Thyroid dysfunction in cerebral venous thrombosis: a retrospective cohort study

**DOI:** 10.1007/s00415-021-10776-3

**Published:** 2021-09-01

**Authors:** Simon Fandler-Höfler, Stefan Pilz, Marion Ertler, Melanie Haidegger, Markus Kneihsl, Gerit Wünsch, Thomas Gary, Christian Enzinger, Thomas Gattringer

**Affiliations:** 1 Department of Neurology, Medical University of Graz, Auenbruggerplatz 22, 8036 Graz, Austria; 2 Division of Endocrinology and Diabetology, Department of Internal Medicine, Medical University of Graz, Graz, Austria; 3 Institute for Medical Informatics, Statistics and Documentation, Medical University of Graz, Graz, Austria; 4 Division of Angiology, Department of Internal Medicine, Medical University of Graz, Graz, Austria; 5 Division of Neuroradiology, Vascular and Interventional Radiology, Department of Radiology, Medical University of Graz, Graz, Austria

**Keywords:** Cerebral venous thrombosis, Thyroid diseases, Hyperthyroidism, Stroke

## Abstract

**Background:**

Cerebral venous thrombosis (CVT) is a multifactorial disease with a variety of related conditions and risk factors. Thyroid dysfunction—especially hyperthyroidism—has been linked to CVT, but this is mainly based on case reports ranging back to 1913, while systematic investigations addressing this issue are lacking. Therefore, we investigated the frequency and clinical characteristics of thyroid dysfunction in a large single-center cohort of CVT patients.

**Methods:**

We retrospectively identified all consecutive patients with aseptic CVT treated at our center between 2006 and 2020. Clinical information was extracted from our electronic medical documentation system. Thyroid-stimulating hormone (TSH) had been routinely measured at admission, free thyroid hormones and thyroid autoantibodies were analyzed whenever available.

**Results:**

Of 120 patients with imaging-confirmed CVT, our main analysis included 107 patients (mean age 42 ± 16 years, 74% female) in whom TSH measurements were available. Nineteen patients (17.8%, 95% confidence interval 10–25%) had thyroid dysfunction. Two had newly diagnosed hyperthyroidism (1.9%, 95% confidence interval 0–4%) caused by Graves’ disease, but without typical symptoms for this condition. Seventeen patients (15.9%, 95% confidence interval 9–23%) had hypothyroidism (12 previously diagnosed with ongoing thyroid hormone replacement therapy; 5 with newly diagnosed subclinical hypothyroidism). Clinical CVT characteristics were similar comparing patients with versus without thyroid dysfunction.

**Conclusion:**

We observed a remarkably high prevalence of thyroid dysfunction in CVT patients. Whether this finding reflects a causal relationship warrants further studies. Despite that, the frequent coexistence of both diseases argues for TSH screening in CVT patients.

## Background

Cerebral venous thrombosis (CVT) is a rare but important subtype of stroke, most frequently affecting women of younger ages [1]. Causes and risk factors for CVT are quite diverse including pregnancy and puerperium, thrombophilia (either heredity or secondary to systemic diseases), prothrombotic medication such as oral contraceptives or hormone replacement therapy and cancer [1].

Hyperthyroidism, most frequently caused by Graves’ disease [2], has the potential to induce a hypercoagulable state and has been associated with venous thrombosis in general [3–5]. The first report linking hyperthyroidism to CVT was published in 1913 [6]. Since then, a substantial number (≥ 30) of case reports have discussed patients with CVT likely caused by hyperthyroidism, with women aged 18–50 years mostly affected [7]. However, systematic studies on the association between hyperthyroidism and CVT are scarce [1].

On the other hand, (untreated) hypothyroidism has also been suggested as a potential cause of CVT in case reports [8], although the pathophysiological foundation for this hypothesis appears much less solid.

Although current literature including reviews on CVT lists “thyroid disease” as a “condition associated with CVT”, cohort studies investigating the frequency and association of thyroid disease in patients with CVT are lacking [1]. As more information regarding this potential association would be useful for clinical management (including the question whether CVT patients should be screened for thyroid dysfunction), we performed a retrospective cohort study investigating the frequency of thyroid dysfunction and associated clinical characteristics in patients with CVT.

## Methods

We investigated all consecutive patients with an imaging-confirmed CVT who were treated at our primary and tertiary university hospital over a fifteen-year-period between 2006 and 2020. Patients were identified using a combination of two search strategies within our electronic hospital information system, by using both ICD-10 codes and free text search in medical/radiological reports. We excluded patients with septic, posttraumatic or postoperative CVT. Septic CVT was defined as related to a local (sinuses, orbital, meningeal) bacterial infection.

**Fig. 1:**
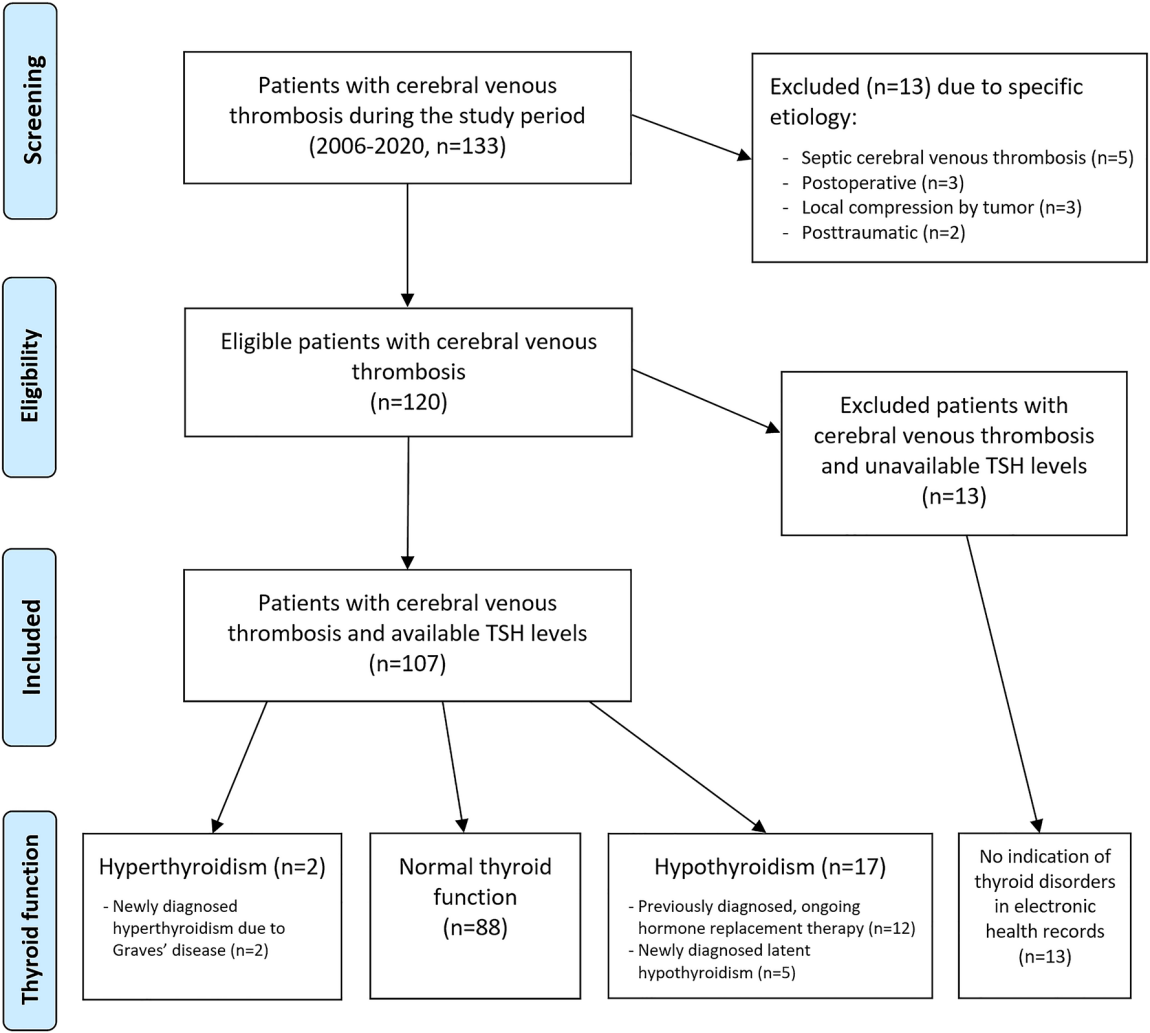
Study flowchart of patient selection and thyroid function

Clinical information including CVT characteristics and risk factors, history of thyroid diseases and medication as well as laboratory data were extracted from our electronic hospital information system (also connecting all public hospitals in our region). Thyroid-stimulating hormone (TSH, normal range: 0.27–4.20 µU/mL) is routinely measured in the majority of patients on the first day of admission to our department (median time from symptom onset to TSH measurement three days, median time from diagnosis to TSH measurement one day). Free thyroxine (fT4) and free Triiodothyronine (fT3) levels were assessed in individual patients in case of TSH concentrations outside the reference range. As we aimed to investigate both clinical and subclinical hyper- and hypothyroidism, we primarily analysed patients with available TSH levels but also screened the remainder for a history of thyroid disease. A flowchart illustrating patient selection is shown in Fig. 1.

According to TSH and fT4 concentrations, we classified our patients as having overt hyperthyroidism (TSH reduced and fT4 elevated), subclinical hyperthyroidism (TSH reduced and fT4 normal), overt hypothyroidism (TSH elevated and fT4 reduced), and subclinical hypothyroidism (TSH elevated and fT4 normal), respectively [9]. Outcome data assessed included mortality, modified Rankin Scale (mRS) scores at discharge and follow-up as well as the rate of recurrent thrombotic events.

## Results

### Patient characteristics

Within the 15-year study period, we identified 120 patients with imaging-confirmed CVT, excluding those with septic, postoperative or posttraumatic origin (Fig. 1). TSH levels were available in 107 patients. In the 13 patients with missing TSH levels, electronic patient records did not indicate preexisting or ongoing thyroid disease or medication. In the entire study cohort, no patient was diagnosed with thyroid cancer before or after CVT diagnosis.

Among the 107 patients with CVT and available TSH values, the mean age was 42.3 ± 16.0 years and 73.8% were female. The most frequent CVT risk factors were the usage of oral contraceptives (35.5%), recent infection (19.6%), either hereditary or secondary thrombophilia (16.8%) and a history of thrombotic events (deep vein thrombosis or pulmonary embolism, 15.9%).

Although 43.9% of patients had parenchymal lesions on neuroimaging and 37.4% were initially treated at the neurointensive care unit, clinical outcome was excellent in the vast majority (84.1% had an mRS score of 0–1 at discharge). Three patients (2.8%) died. During a mean follow-up period of 16 months (range 2–126 months), only one patient had a recurrent CVT, while five patients had other recurrent venous thrombotic events. Clinical characteristics of the study cohort are shown in Table 1.

**Table 1:** Clinical characteristics of the entire study cohort and in patients with previously and newly diagnosed hypothyroidism

	Study cohort	Hypothyroidism	
	*n* = 107	Previously diagnosed (*n* = 12)	Newly diagnosed (*n* = 5)
Demographic data			
Age (years), mean ± SD	42.3 ± 16.0	44.4 ± 14.9	28.6 ± 9.6
Female sex	79 (73.8%)	11 (91.6%)	5 (100.0%)
Clinical symptoms			
Headache	88 (82.2%)	12 (100%)	3 (60.0%)
Reduced visual acuity	13 (12.1%)	2 (16.7%)	1 (20.0%)
Seizure	33 (30.8%)	4 (33.3%)	2 (40.0%)
Focal-neurological deficits	34 (31.8%)	5 (41.7%)	1 (20.0%)
Risk factors			
Thrombophilia (hereditary/acquired)^a^	18 (16.8%)	2 (16.7%)	0
Systemic autoimmune disease	11 (10.3%)	3 (25.0%)	0
Pregnancy/puerperium	8 (7.5%)	1 (8.3%)	1 (20.0%)
Cancer	8 (7.5%)	1 (8.3%)	0
Previous venous thrombotic event	17 (15.9%)	4 (33.3%)	1 (20.0%)
Recent infection	21 (19.6%)	3 (25.0%)	0
Oral contraceptives	38 (35.5%)	4 (33.3%)	3 (60.0%)
Other prothrombotic medication	12 (11.2%)	1 (8.3%)	1 (20.0%)
Imaging Data			
MRI performed	96 (89.7%)	11 (91.6%)	5 (100%)
Parenchymal lesions	47 (43.9%)	5 (41.7%)	2 (40.0%)
Lateral sinus system affected	90 (84.1%)	10 (83.3%)	5 (100%)
Superior sagittal sinus affected	46 (43.0%)	5 (41.7%)	2 (40.0%)
Deep veins affected	28 (26.2%)	0	0
Cortical veins affected	21 (19.6%)	4 (33.3%)	2 (40.0%)
Thyroid hormone levels			
Thyroid-stimulating hormone (µU/mL)	1.94 ± 13.0	1.35 ± 1.06	5.45 ± 0.73
Free thyroxine (pmol/L, mean ± SD)^#^	16.6 ± 10.8	16.48 ± 4.26	14.88 ± 3.42
Free triiodothyronine (pmol/L, mean ± SD)^#^	4.92 ± 2.8	3.38 ± 0.53	4.57 ± 0.80
Clinical course			
Neurointensive care unit treatment	40 (37.4%)	4 (33.3%)	1 (20.0%)
Median modified Rankin Scale at discharge	0 (range 0–6)	0 (range 0–2)	1 (range 0–2)
In-hospital mortality	3 (2.8%)	0	0
Recurrent venous thrombosis	5 (4.5%)	1 (8.3%)	0
Recurrent cerebral venous thrombosis	1 (0.9%)	1 (8.3%)	0

^a^Factor V Leiden mutation (*n* = 7), Prothrombin mutation (*n* = 4), Antiphospholipid antibody syndrome (*n* = 3), MTHFR mutation (*n* = 2), Protein S deficiency (*n* = 1), Thrombocythemia (*n* = 1)

^#^Available in 33/107 patients

### Hyperthyroidism

Among the 107 patients with available TSH levels, we identified two patients (1.9%, 95% confidence interval: 0–4%) with newly diagnosed overt hyperthyroidism. Both patients had no hyperthyroidism-related symptoms but instead were detected on the basis of routinely obtained (suppressed) TSH, and subsequent work-up showed elevated fT3 and fT4. Graves’ disease was the cause of hyperthyroidism in both cases as evidenced by elevated TSH receptor antibodies (TRAbs).

The first patient was a 60-year-old female with isolated left-sided headache who had a CVT in the left transverse and sigmoid sinus with associated left temporoparietal intracerebral haemorrhage. Aside from the newly diagnosed hyperthyroidism, no other CVT risk factors were identified. The patient received low-molecular-weight heparin followed by dabigatran for CVT and methimazole for hyperthyroidism and was discharged with no residual symptoms (mRS 0). The second patient with CVT and hyperthyroidism was a 35-year-old female who also had isolated headache. MRI confirmed CVT in the deep cerebral venous system including the straight sinus, left-sided internal cerebral vein, left transverse sinus and jugular vein. Prothrombotic risk factors included the usage of oral contraceptives. Low-molecular-weight heparin and methimazole were initiated. She was also discharged with an excellent outcome (mRS 0). In both patients, no recurrent venous thrombotic events occurred during the entire follow-up period.

### Hypothyroidism

Seventeen patients (15.9%, 95% confidence interval: 9–23%) in our cohort had hypothyroidism (Table 1). In 12 of them, hypothyroidism was diagnosed prior to CVT and all were treated with thyroid hormone replacement at the time of CVT diagnosis (TSH, fT3 and fT4 values were all normal). In the other five patients, we detected subclinical hypothyroidism (elevated TSH values with normal fT3/fT4 levels). During follow-up examinations, Hashimoto’s thyroiditis was diagnosed in two of these patients with thyroid hormone replacement initiated, while we lack thyroid-specific follow-up information in the three other patients. CVT patients with hypothyroidism were more often female and those with newly diagnosed hypothyroidism were younger, other clinical characteristics and risk factors were comparable between patients with versus without hypothyroidism (Table 1).

A graphical summary of thyroid function in our study cohort can be found in Fig. 1.

## Discussion

In this monocentric cohort study, we found thyroid dysfunction in 19 out of 107 patients (17.8%)—hyperthyroidism in two patients and hypothyroidism in 17 patients. To the best of our knowledge, there is only one prior systematic investigation on this topic. A recent German single-centre study on 182 patients with CVT (identified over a 20-year-period) found a frequency of current thyroid disease (at the time of CVT) of 11% and previous thyroid disease of 9.9% [10]. Although these numbers were unexpectedly high, thyroid hormone levels were not systematically investigated in that study cohort, which may have led to an underdiagnosis of subclinical thyroid dysfunction. In contrast, we here systematically investigated the prevalence and clinical characteristics of thyroid dysfunction in patients with CVT including a systematic evaluation of thyroid hormone levels, which enabled us to identify all potential patients with subclinical thyroid dysfunction.

In the International Study on Cerebral Vein and Dural Sinus Thrombosis (ISCVT), a multinational study of 624 patients with CVT, *“thyroid disease”* was reported in 1.7% of patients, but the authors did not specify the exact nature or definition of thyroid disease and it was not specifically screened for [11]. This number has been used since, including current reviews on CVT [1]. Although a direct comparison with our study is also hampered by the different study settings (including geographical differences), we found a much higher prevalence of thyroid dysfunction in CVT patients. The rate of overt hyperthyroidism (1.9%, newly diagnosed) in our study cohort exceeds prevalence estimates in the general population of 0.3–0.6% for overt hyperthyroidism [12, 13] and 1–1.5% for Graves´ disease [12]. Furthermore, the overall rate of thyroid dysfunction (17.8%) in our study cohort clearly exceeds prevalence estimates from a European meta-analysis in the general population (3.8%) [13].

Notably, both patients with CVT and newly diagnosed hyperthyroidism (due to Graves’ disease) did not have thyroid-related symptoms but were detected by routinely obtained (suppressed) TSH values leading to further laboratory and clinical work-up. Although we cannot conclude on causal inference between hyperthyroidism and CVT, there is solid evidence linking hyperthyroidism to an increased risk of venous thrombosis in general [3–5, 14] and numerous case reports have reported an association between hyperthyroidism and CVT [7].

Although the rate of hypothyroidism was surprisingly high in this CVT cohort, all 17 affected patients had normal peripheral thyroid hormone levels (12 patients with previously diagnosed hypothyroidism and ongoing hormone replacement therapy; and 5 patients with newly diagnosed subclinical hypothyroidism). As thyroid-specific follow-up data is missing on three of these five patients, we cannot clearly conclude whether subclinical hypothyroidism was transient or persistent in those three patients. The prevalence of newly diagnosed subclinical hypothyroidism (4.7%) is very similar to a recent meta-analysis of undiagnosed hypothyroidism in Europe (4.1% for subclinical hypothyroidism and 4.7% for any hypothyroidism) [15], but the rather large overall prevalence of patients with hypothyroidism including also previously diagnosed hypothyroidism significantly exceeds population estimates of up to 5% [13]. Although one study describing two cases has suggested an association between hypothyroidism and CVT [8], hypothyroidism has not been related to a higher rate of venous thrombosis, with multiple studies pointing even towards a hypocoagulative state in patients with overt hypothyroidism [3, 14].

Limitations of our study include those related to the retrospective single-centre design. Our study design allows only to describe the associations found as reported, no causality can be drawn from our results. Furthermore, the study lacks a control group and we are well aware that a direct comparison to population-based studies is limited as our results may also be driven by the specific demographic and clinical characteristics of our study cohort. The frequency of thyroid diseases may vary in different geographical locations due to differences in iodine nutrition [16], therefore our finding in a Central European population may not be directly translatable to iodine-deficient regions. Furthermore, fT3 and fT4 values were not obtained in all patients, but significant thyroid dysfunction would have been detected through the assessment of TSH levels.

In conclusion, we observed a relatively high prevalence of thyroid dysfunction (both hyper- and hypothyroidism) in patients with CVT warranting further investigations to confirm our findings, to better disentangle the designation “thyroid dysfunction” as used in the ICVST and subsequent reviews, and to ideally elucidate the underlying mechanisms for these associations.

## Data Availability

The datasets generated during this study are available from the corresponding author upon reasonable request.
